# Hyperthyroidism–cause of depression and psychosis: a case report

**Published:** 2009-11-25

**Authors:** G Marian, AE Nica, BE Ionescu, D Ghinea

**Affiliations:** * UMF Carol Davila, BucharestRomania; ** Clinical Hospital ‘Prof. Dr. Al. Obregia’, BucharestRomania

**Keywords:** hyperthyroidism, psychosis

## Abstract

Psychiatric symptoms have been reported quite frequently in certain thyroid diseases, but more frequently in association with hypothyroidism. Thyrotoxicosis can be associated with various psychiatric symptoms, such as emotional lability, anxiety, restlessness and rarely frank psychosis. Psychotic symptoms in the context of hyperthyroidism typically present as an affective psychosis. The link between psychosis and hyperthyroidism is poorly understood. Because of this association of psychiatric symptoms is important to exclude a somatic cause, when assessing a patient first.

We present the case of young woman who was followed over 2 years and who initially presented to psychiatric consultation for depressive symptoms, after being diagnosed with hyperthyroidism and specific therapy instituted, but who developed psychotic symptoms.

## Introduction

Hyperthyroidism is frequently associated with: irritability, insomnia, anxiety, restlessness, fatigue, impairment in concentrating and memory, these symptoms can be episodic or may develop into mania, depression and delirium. In some cases motor inhibition and apathy are symptoms that accompany hyperthyroidism.

Psychosis is a rare complication in hyperthyroidism, it was reported in 1% of cases and most patients who develop psychosis have been previously diagnosed with mania and/or delirium [1]. The fact that certain psychotic symptoms can be found in patient with hyperthyroidism, which was in its specific treatment for the disease raises both diagnostic and therapeutic problems and collaboration between specialists is necessary to clarify the cause of symptoms and to establish an appropriate treatment as soon as possible.

## Case presentation

We present a case of a female patient (38 years old), without psychiatric history who was observed over 2 years (2007–2009). Patient is nonsmoker, no drugs abuse and there is no family history of psychiatric disorders.

***May 2007***: the patient presents to the psychiatric ambulatory for depressive symptoms (depressed mood, crying easily, depressive thought of hopelessness, drop out of social activities, slowed thinking, speech and body movements, fatigue) with no clinical or biological sign for a somatic disease. It was established diagnosis of major depressive episode for which was instituted treatment with Fluoxetine 20mg per day. The treatment was followed for 2 month without a significant improvement of symptoms.

***July 2007***: patient shall be submitted for review therapy; when examining patient had: depressed mood, crying easily, tremor, palpitations, minor exophthalmos, weight loss of 3 kilograms. The dose of Fluoxetine was increase to 40 mg per day. In this context was require endocrine evaluation. Blood analysis was performed with the following results: blood count and leukocyte formula in normal ranges, glucose in normal range, hepatic tests normal, normal urine sediment. The result of thyroidian tests was: T3= 500ng/dl (normal parameters between 70–210ng/dl), FT4= 20ng/dl (normal parameters between 0.7–2ng/dl) and TSH=0.01microU/ml (normal parameters between 0.3– 6.2microU/ml). After an evaluation is established diagnosis of hyperthyroidism and establish treatment with Propiltiouracil 600mg per day Treatment was maintained 3 months.

In ***October*** the patient is brought to the psychiatric emergency room for a delirious hallucinatory symptoms (extraordinary irritability, visual and auditory hallucinations, delusions of persecution and pursuit, psychomotor agitation, stereotype behaviour). Somatic examination found: marked bilateral exophthalmos, hyperhidrosis, tremor of extremities, goiter, tachycardia, vomiting.

At this point we decided to interrupt antidepressant treatment and to maintain therapy with Propiltiouracil 600mg per day. The result of thyroidian tests was: T3= 800ng/dl (normal parameters between 70–210ng/dl), FT4= 27ng/dl (normal parameters between 0.7–l2ng/dl) and TSH=0.002microU/ml (normal parameters between 0.3–6.2microU/ml). With all this, the diagnosis of thyrotoxicosis is suspected. The term of thyrotoxicosis refers to clinical, psysiological and biochemical manifestations that occur after exposure and response of the tissues to the excessive supply of thyroid hormone [2]. It was established the diagnose of acute psychotic episode with symptoms of schizophrenia and patient received treatment with Risperidone 4mg per day. After 6 weeks delirious hallucinatory symptoms were resolved incomplete (hallucinations disappeared and the patient began to communicate verbally, but unstable delusions still remain). Interdisciplinary consultation recommended thyroid excision as therapeutic approach. The patient was prepared psychologically for the accomplishment of total thyroidectomy with full acceptance (it was maintained treatment with risperidone 4 mg per day).

***January 2008***: thyroidectomy was performed and establish replacement therapy with levothyroxine ( 125microg per day). Within 3 months thyroid tests were normalized and was dropped antipsychotic treatment (the dose was gradually reduced to that with normalization of thyroid tests to give to antipsychotic treatment).

Psychiatric reassessment has highlighted the temporal correlation between psychotic symptoms and decompensation of thyroidian status. Final psychiatric diagnosis was that the psychotic episode due to general medical condition (thyrotoxicosis).

## Problems of diagnosis and treatment

This clinical case has questioned the diagnosis and treatment both in terms of psychiatric and endocrine conditions:

1. Lack of response to antidepressant treatment corroborated with clinical signs of hyperthyroidism oriented identification of underlying medical causes, that why the first and most important step in the assessment of a patient with psychiatric symptoms is to eliminate the somatic causes (3).
2. The efficiency of oral medication with Propiltiouracil proved to be less than expected, the psychiatric symptoms evolving towards a full blown psychotic episode; a differential diagnosis between an acute psychotic episode with schizophrenia symptoms and one induced by a general medical condition must be made.
  Aspects that suggest an organic origin of the behavior disorders (4):
  - some atypical characteristics for a specific psychiatric diagnosis;
  - behavior symptoms that is more spectacular than that expected according to the isolated psychiatric syndrome;
  - late onset age in a new behavioral symptom;
  - absence of personal or familial history of psychiatric disease;
  - sudden onset of psychosis or dementia;
  - poisoning or withdrawal of some substance, or the prescription of substances with psychoactive properties (i.e. digoxin);
  - obvious systemic disease;
  - evidence of increase of intracranial pressure;
  - non–auditory hallucinations (visual, distortions and illusions are the most suggestive);
  - time relationship between physical state and onset, exacerbation and end of psychiatric symptoms;
  - appearance of autonomic dysfunction without there being pre–morbid dysfunction;
  - resistance to psychiatric treatment;
  - alterations of consciousness level, orientation or memory.
3. Endocrine reevaluation is required due to the lack of pharmacological control of thyroid disease. Surgical ablation of thyroid decision allowed such control of endocrine disease. The disappearance of psychotic phenomena, consecutive to normalization of thyroid tests led to antipsychotic discontinuation secure.

## Conclusions

Thyroid disease should be considered in the differential diagnosis of a large spectrum of psychiatric symptoms; early treatment of the hormone or metabolic alteration can minimize the morbidity of a secondary psychopathology. In addition, in most of the patients, it is enough to correct the psychiatric symptoms, although, in a subgroup of them, the resolution of some pictures, such as depression, may not occur after the treatment of the endocrine or metabolic dysfunction and may require specific psychiatric treatment. Even so, psychiatric symptoms can dynamically changed into a veritable psychosis and antipsychotic agents may be necessary to control the hallucinatory delirious symptoms (5). Our case became more difficult because of  initial endocrine and psychiatric treatment failure which led to a radical solution: thyroidectomy. The benefit of this approach was immediately observed with full control of both conditions.
